# Dielectrophoretic Immobilization of Yeast Cells Using CMOS Integrated Microfluidics

**DOI:** 10.3390/mi11050501

**Published:** 2020-05-15

**Authors:** Honeyeh Matbaechi Ettehad, Pouya Soltani Zarrin, Ralph Hölzel, Christian Wenger

**Affiliations:** 1 IHP–Leibniz-Institut für innovative Mikroelektronik, Im Technologiepark 25, 15236 Frankfurt/Oder, Germany; soltani@ihp-microelectronics.com (P.S.Z.); wenger@ihp-microelectronics.com (C.W.); 2 Fraunhofer Institute for Cell Therapy and Immunology, Branch Bioanalytics and Bioprocesses (IZI-BB), 14476 Potsdam-Golm, Germany; Ralph.Hoelzel@izi-bb.fraunhofer.de; 3 BTU Cottbus-Senftenberg, 03046 Cottbus, Germany

**Keywords:** dielectrophoresis, cell immobilization, cell separation, interdigitated electrodes, microfluidics, lab-on-a-chip

## Abstract

This paper presents a dielectrophoretic system for the immobilization and separation of live and dead cells. Dielectrophoresis (DEP) is a promising and efficient investigation technique for the development of novel lab-on-a-chip devices, which characterizes cells or particles based on their intrinsic and physical properties. Using this method, specific cells can be isolated from their medium carrier or the mixture of cell suspensions (e.g., separation of viable cells from non-viable cells). Main advantages of this method, which makes it favorable for disease (blood) analysis and diagnostic applications are, the preservation of the cell properties during measurements, label-free cell identification, and low set up cost. In this study, we validated the capability of complementary metal-oxide-semiconductor (CMOS) integrated microfluidic devices for the manipulation and characterization of live and dead yeast cells using dielectrophoretic forces. This approach successfully trapped live yeast cells and purified them from dead cells. Numerical simulations based on a two-layer model for yeast cells flowing in the channel were used to predict the trajectories of the cells with respect to their dielectric properties, varying excitation voltage, and frequency.

## 1. Introduction

Cell characterization and manipulation are critical when it comes to clinical and diagnostic applications [1]. Immobilization and isolation of specific cells as a way to detect diseases [2,3,4], separation of live and dead cells as a means for early-stage disease diagnosis [5,6], as well as filtering and purification of cells, viruses, proteins, and micro/nanoparticles [7,8,9,10] are essential examples in a variety of biological and biomedical applications. Development of lab-on-a-chip (LOC) devices such as microfluidic platforms has simplified the handling of complex and costly laboratory-based sample preparations and analyses, using a single device in the scale of a few centimeters [8,9,10]. Performing various tasks on a single device not only increases the precision of analysis but also improves the accuracy, reliability, and reproducibility of sample preparation procedure. 

Among various cell manipulation techniques for LOC devices, dielectrophoresis (DEP) has been utilized widely for biomedical applications [11,12]. DEP is a non-invasive, label-free, and low-cost method which provides high accuracy and efficiency analyses [11]. Since this method exploits the intrinsic dielectric properties (relative permittivity and electrical conductivity) of the cells and their surrounding medium [12], it can be used selectively for the characterization and manipulation of cells. When polarizable cells subject to a non-uniform AC electric field, DEP force is induced as a result of the interaction between the cells’ induced dipole and the electric field [13]. A non-uniform electric field can be generated as a result of imposing an AC signal to an electrode. Variation in the frequency of the applied signal can generate DEP forces in two opposite directions, resulting in either positive DEP (pDEP) or negative DEP (nDEP). Based on the selective DEP forces, specific cells can be trapped and detected [2,4,14], collected for further analyses (e.g., viability test) [15,16], or isolated from a mixture of cell suspension in blood for purifying processes [17]. Furthermore, dead cells, which cause bias during experimental measurements, can be removed from live cells [18]. 

Planar [18,19,20] and three-dimensional (3D) electrode structures [21,22] are commonly used for these applications. 3D electrodes are fabricated on the top and bottom, or sidewalls of microfluidic channels, whereas planar electrodes are commonly embedded on the bottom of microfluidic channels [23]. Prominent examples of planar electrodes are, interdigitated electrode arrays (IDEs), castellated [24], quadrupole [25], curved [26], spiral [1], oblique [27] and matrix [28]. Among these planar electrodes, IDEs are the convenient form of electrode geometry for dielectrophoretic immobilization [29] and separation [8] of certain cell population. IDEs have been previously used to immobilize biological entities [6], proteins [30], and to detect the dielectric constant of organic fluids [31], etc.

Over recent decades, many studies have been conducted on characterization and detection of the biological species on a single chip. Flanagan et al. [32] explored the use of DEP for characterization and identification of stem cells and their differentiated progeny. To create DEP force, IDEs were fabricated on glass wafers and were placed at the bottom of a polymer-based microfluidic channel. DEP showed that stem cells and their differentiated deviations develop different dielectric properties. Although this approach presented a platform to distinguish specific cells, it employs a large-scale setup. Lyu et al. [33] presented a numerical model using COMSOL simulations on the development of an electroporation technology for simultaneously calculating the DEP forces and electroporation of yeast and E.coli cells in the fluid flowing on a non-electrolytic micro/nano electroporation (NEME) electrode surface. Although this advancement could lead to new medical applications such as cell separation and destruction of unwanted cells, the applicability of this method has not been validated and confirmed experimentally. Ning et al. [34] described a test system for simultaneous microwave measurement and visual validation of cytoplasm resistance of a live Jurkat using broadband electrical detection technique. The setup is based on a homemade probe station mounted on top of an inverted microscope. This system included gold-based coplanar waveguide (CPW) placed between a quartz substrate and a PDMS cover. A 150 µm wide and 50 µm height channel etched underside of the PDMS cover. In other work, Li et al. [35] proposed a similar microfluidic setup to differentiate between the small number of live and heat-killed Escherichia coli cells suspended in culture media using microwave measurement. The differentiation principle between live and dead cells is based on the comparison of the transmitted and reflected microwave signals. The off-chip analysis showed that the difference is due to the decrease of cytoplasmic dielectric properties over cell death. A bipolar complementary metal-oxide-semiconductor (BiCMOS) based LOC platform was proposed by Manczak et al. [36] for discrimination of Glioblastoma (GBM), undifferentiated from differentiated cells, using ultra-high frequency (UHF) DEP technique to characterize cancer stem cells. Using this technique, characterization and detection of cells were achieved based on the intracellular dielectric properties of individual cells. To avoid the contact of cell population suspended in a liquid carrier with electronics of the chip, a polydimethylsiloxane (PDMS) microfluidic channel fabricated above the sensors on top of the BiCMOS device. All of these published methods offer opportunities to characterize and detect bio-particles on the same chip. However, they utilized relatively large-scale setups with polymer-based microfluidic channels that are not compatible with complementary metal-oxide-semiconductor (CMOS) process flows. The bulky polymer-based LOC setups limit the device performance by introducing parasites to the system. Moreover, PDMS microfluidics is more convenient for lab-based researches than for industrial applications because of the limited reproducibility of the fabrication process [37]. Among many alternative materials, such as polymethylmethacrylate (PMMA), amorphous polymers, thermoplastics and epoxy photoresist SU-8 [38], silicon is a reliable substitute for polymer in microfluidic applications due to its high integration robustness with electronics. Furthermore, silicon can be used in conjunction with fluidic applications which requires, high temperature resilience, very high precision channel alignments and high aspect ratio structures [38]. One of the most outstanding advantageous of silicon is the possibility of fabricating thin membranes which improves the stability of the device to high temperature ramp-rates by reducing thermal mass [38,39]. High thermal conductivity of the silicon warrant a uniform temperature distribution [40]. Thus, on the one hand, the interest in using silicon-based microfluidic LOC is increasing. On the other hand, the need for physically interfacing the fluidic samples with electrodes and sensors for analyzing biological and nonbiological samples is increasing the demand for combing the capabilities of microfluidics and CMOS integrated circuits. Integrating these technologies provides remarkable opportunities in the biomedical field for point-of-care diagnostics, high throughput screening, and implantable devices [41]. The hetero-integrated CMOS technology allows the fabrication of microfluidic channel, sensors, and circuitry as monolithic devices. Due to the fact, that trapping, sensing, detecting and analyzing can be achieved on a single chip, the hetero-integrated CMOS approach is very beneficial for future applications, while polymeric-based microfluidic channel approaches lacks from sensor and circuitry integration on a single chip solution. Combination of CMOS and microfluidics on the same die allows highly miniaturized LOC fabrication. Moreover, the high alignment accuracy of CMOS processing enables smaller distances between the isolated fluidic and electrical interfaces. Integrating microfluidics process steps into CMOS fabrication for miniaturized microsystems not only facilitate the LOC portability, but also enable fast diagnostic results even under non-laboratory conditions. However, lack of promising integration methods remains a big a challenge and realizing a fully functional device is under research.

In this paper, we investigated a 5 × 5 mm² CMOS integrated silicon microfluidic device utilizing six various IDEs, with different geometrical ratios, for the immobilization and separation of live and dead yeast cells using dielectrophoresis. The idea of combining the fluidic solution and electrical components improves the functionality and precision of this highly miniaturize LOC by using separate interfaces for electrical connections and microfluidics. This approach provides a low voltage DEP technique and an operational simplicity that enables the portability of the LOC device. The hetero-integration technology which allows the fabrication of microfluidic channels, sensors, and circuitry on a single chip, is replaced by the costly multi-step fabrication processes of various chips. The high alignment accuracy of the microfluidic channel on CMOS electronics ensures a reproducible and reliable integration process compared to relatively large size polymeric-based microfluidic LOC systems. The opportunity of immobilizing, sensing, and detecting cells on the same chip increases the reproducibility of the measurements by using less complex setups. Contamination-free fabrication process of CMOS integrated microfluidic offers reliable measurements. Using silicon instead of polymer for the fabrication of the microfluidic channel benefits the high integration level of circuitry and sensors on a single chip. State-of-the-art of this CMOS technology offers the opportunity of immobilizing, sensing, and detecting the particles on the same chip. However, sensing and detecting of the cells are not in the scope of this article and will be described in details elsewhere. The IDE structures used in this study followed the Guha. et al. approach, which used a similar structure for sensing and detecting biological cells on a single chip [42,43,44,45]. To optimize and adapt this IDE to our application, a systematic simulation study was conducted using COMSOL Multiphysics (version 5.3) [46,47]. A wide range of different electrodes with varying electrode width and spacing between fingers were modeled. To confirm the simulation results, some promising structures were selected and proposed for fabrication. Two main concepts have been scrutinized and demonstrated throughout this paper, which include the applicability of dielectrophoresis for cell immobilization and the impact of voltage, frequency, flow rate, and geometry ratio (spacing to width) of various IDEs on the time-dependent DEP behavior of live yeast cells suspended in deionized (DI) water. The choice of yeast cell as a model organism and DI-water as a model liquid carrier was done to keep the first model as simple as possible and reduce the number of complex parameters to obtain a trustable comparison between simulation and experimental results. However, this device can also be used for the analysis of cells suspended in more complex mediums. The cells motion were investigated optically and compared with the simulation results. Moreover, this paper proposes the adaption of the developed LOC device for the isolation and separation of viable and non-viable yeast cells in a mixture. 

## 2. Materials and Methods 

### 2.1. Microsystem

The LOC platform introduced in this work [48] combines a microfluidic channel with high-performance CMOS electronics. Based on this technique, separate microfluidic and electrical interfaces can be achieved. The developed silicon microfluidic channel is integrated into a CMOS device and encapsulated with transparent glass for simultaneous electrical and optical measurements. The combination of microfluidics and CMOS technologies offers great benefits in terms of high throughput integration level and cost reduction, thus making the approach favorable for biomedical applications. By taking advantage of the system miniaturization, designing small-sized channels and integrating sensors near the fluidic interface are possible, which ultimately leads to a higher sensitivity of the LOC system. 

### 2.2. Microfluidics

**Figure 1:**
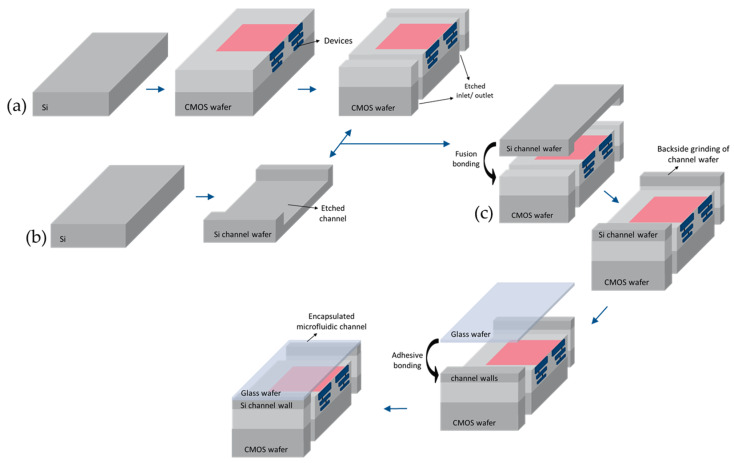
Schematic cross-sectional view of the microfluidics lab-on-a-chip (three-wafer stack) packaging process [48]: (**a**) CMOS fabrication; (**b**) Formation of the microfluidic channel; (**c**) Three-wafer-stack approach bonding process.

The state-of-the-art of our LOC device is due to the hetero-integration of the microfluidics and compatibility of the in-house CMOS technology with the standard processing technology. It is noteworthy that the fabrication cost reduction, reproducibility and reliability are the main benefit of this approach. CMOS electronics, Si channels, and the glass wafer are integrated (a three-wafer-stack approach) on a single chip using 200 mm wafer bonding techniques [48]. Figure 1 illustrates the fabrication process of the microfluidics LOC device. In this process, the first wafer used to fabricate the CMOS device, including active circuitry and sensors. Next, the inlet and outlet for the microfluidic channel were opened by Localize backside etching (LBE) from the backside of this wafer. The second bare Si wafer is patterned to structure the channel by etching. Using plasma-activated oxide-oxide fusion bonding, these wafers are bonded together from their front sides at 300 °C. This step is followed by grinding the backside of the microfluidic channel to achieve the desired channel height. Finally, to seal the microfluidic channel, the third wafer, which is a glass wafer, is adhesively bonded to the top of the channel at 200 °C. 

The reduced silicon-based channel dimensions (low channel height) compared to the polymeric-based fluidic channel with relatively larger sizes, increase the chance of bringing cells closer to the fringing electric field created by the IDEs. Larger channels increase the probability of cell tracing from above the effective distance of fringing field over IDEs which results in the discard of cells from the channel. Development of the current device satisfy the need of a more reliable analyzation of small sample amounts.

### 2.3. Interdigitated Electrodes 

**Figure 2:**
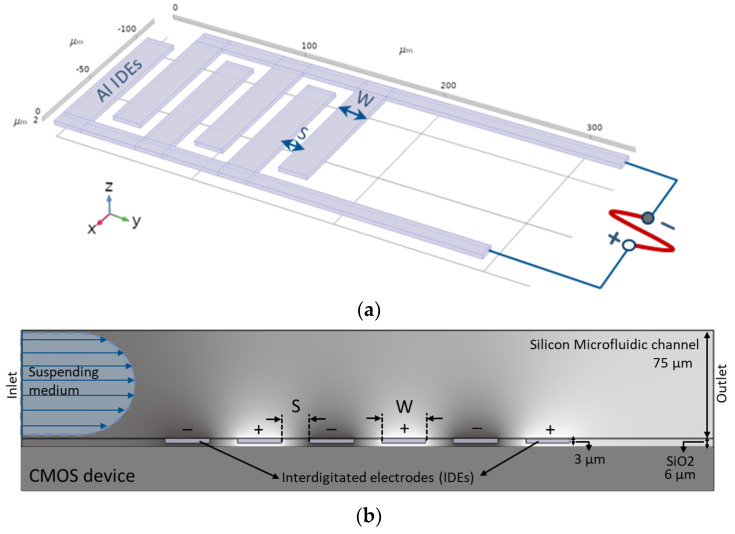
(**a**) Multi-fingered planar IDEs, (**b**) Cross-sectional view of IDEs embedded in the microfluidic channel.

Arrays of microfabricated IDEs are the convenient form of electrode geometry for the dielectrophoretic characterization of biological particles (e.g. cells and viruses), through microfluidic biochips. In this work, a multi-fingered planar IDE is used as electrodes (Figure 2a) and embedded in the microfluidic channel (Figure 2b). 

**Figure 3:**
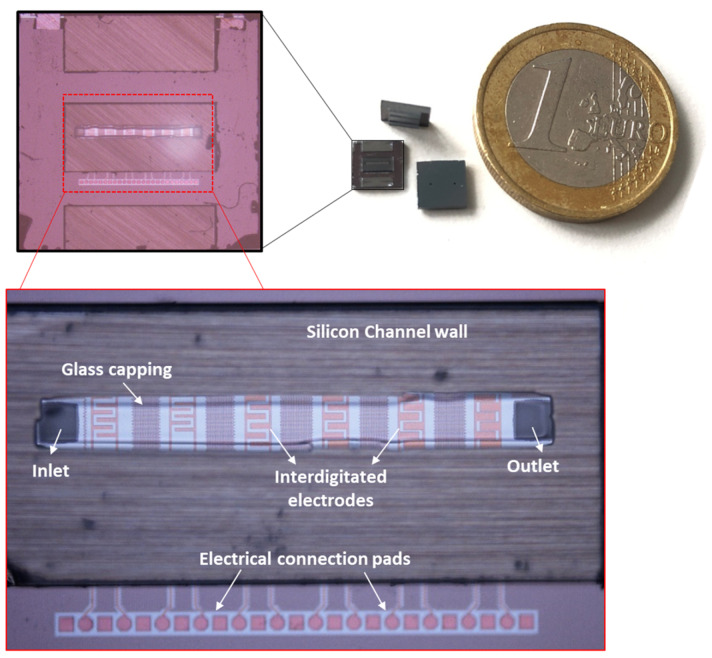
CMOS integrated microfluidic lab-on-a-chip devices with zoom-in of the chip and embedded IDEs in the channel.

These electrodes are used for the separation of particles or purification of the live cells from dead cells. In this work, we initially used the same IDE structures which were previously established for high-frequency CMOS dielectric sensors [49]. However, these IDEs were then geometrically optimized to enhance the DEP performance, using COMSOL simulations [47]. Various IDEs with different geometrical parameters were simulated. To confirm and validate the simulation results experimentally, various IDEs were fabricated. For the first prototype of the CMOS integrated microfluidic channel, due to design limitations for electrical contacts, these IDE structures were fabricated perpendicularly to the microfluidic channel. Table 1 represents the geometrical parameters of the manufactured IDE structures [47]. IDE structures were fabricated in the standard 0.25 µm CMOS technology of IHP. Figure 3 illustrates the device chip. The commonly used CMOS compatible material chosen to fabricate the IDEs are known to be long term stable in CMOS based products. The reliability issues of the same material used for biochip fabrication have to be evaluated in the future.

### 2.4. Cells under Test

Yeast cells (Saccharomyces cerevisiae RXII) were used for DEP studies as the model particles. Live yeasts were diluted in 40 mL deionized (DI) water at a concentration of 15×10^2^ µg mL^−1^ and incubated at room temperature for 15 min and were stirred every 5 min. Dead cells obtained by heating live cell suspension in DI-water with the same concentration, at 100 °C for 20 min, and mixed with live ones for the separation experiment. The average diameters of the live and dead cells were measured as 8 µm and 6 µm, respectively. Sample suspensions were introduced into the microfluidic chip using a syringe pump.

### 2.5. Experimental Setup

**Figure 4:**
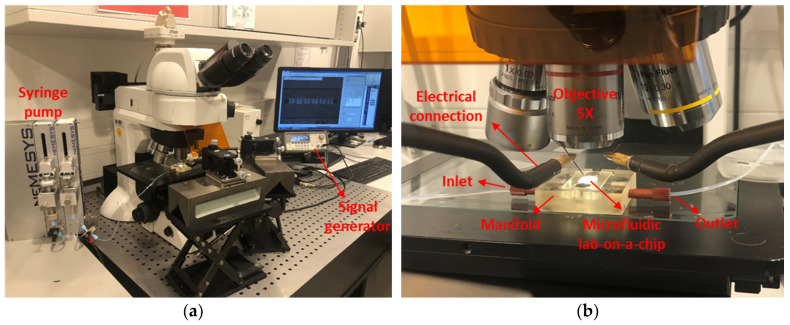
Experimental setup: (**a**) equipment used for dielectrophoresis characterization of yeast cells; (**b**) lab-on-a-chip with electrical connections under the objective.

Figure 4 presents our experimental setup, which consists of an AC signal generator (Agilent-33220A, Agilent Technologies/Keysight Technologies, Santa Clara, CA, USA) to generate a fringing electric field between the IDE fingers, the programmable syringe pump (NEMESYS, CETONI GmbH, Korbußen, Germany) to flow the cells which are suspended in DI-water, a tabletop, and an upright microscope (Nikon Eclipse-LV100ND, Nikon GmbH, Tokyo, Japan) equipped with a CCD video camera (Nikon-DS-Fi2, Nikon GmbH, Tokyo, Japan) connected to a computer for simultaneous optical measurement and analysis of the acquired videos and images.

**Figure 5:**
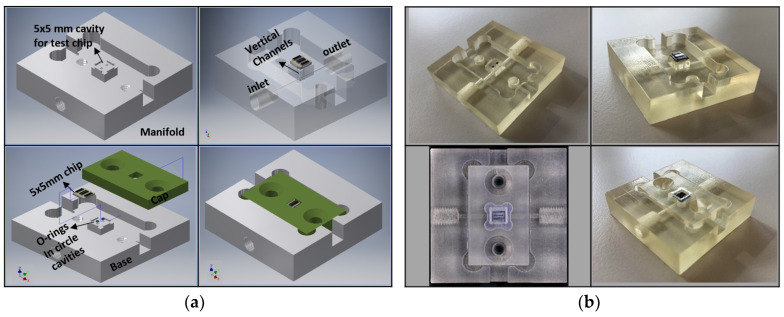
Manifold technology development using 3D printing for holding the lab-on-a-chip: (**a**) 3D schematic of the manifold design; (**b**) Fabricated manifold using 3D printing including a test chip.

To provide an interface to control the fluid flow and sample injection through the microfluidic channel, an external macrofluidic technology was employed. To this end, a fluidic manifold was designed, Figure 5a, and fabricated out of transparent hard polymer (PMMA) using a commercial 3D printer (Keyence Agilista-3200W, Keyence Co., Osaka, Japan), Figure 5b, interfacing the micro-device to the macroscale fluidic connections. A cavity with the same size as the chip, 5 × 5 mm², is created in the manifold. The square-shaped inlet and outlet (with the dimension of 150 µm) of the microfluidics were aligned directly on the manifold channels from the bottom side of the CMOS chip. At the interface of the chip and manifold, two O-rings with an inner diameter of 0.5 mm were used to seal the fluidic connections between the manifold and the chip to prevent leakage. The chip is clamped between the fluidic manifold base and cap by two screws. The external tubing connections were made via thread connectors, which are screwed into the inlet/outlet ports of the manifold.

The dielectrophoretic characterization and immobilization of yeast cells in the microfluidic channel was attained by imposing the AC signal (electric field) across the IDEs. Six IDE structures with varied ratios of spacing to width were used separately to conduct DEP characterization studies on yeast cells. A signal generator supplied the electric field. The AC signal frequency was varied from 1 kHz to 20 MHz (limited by the signal generator with the maximum output voltage of 20 V). The syringe pump loaded the cell suspension into the chip. Yeast suspensions were driven through the channel with a flow rate of 50 µm s^−1^. When the cells reached the region of the IDEs, the flow was stopped, and when the cells were settled (after 15 s), then the flow rate was increased to 1 µm s^−1^ and was kept constant during the DEP immobilization. Initiation of the high flow rate fluid through the channel results in discards of the cells from the channel rather than immobilization and entrapment of the cells to the IDEs. Disconnecting the AC signal from the electrodes after cell immobilization results in desorption of the entire entrapped cells from the IDEs and removing the cells from the channel. Cells trajectory were observed under the microscope for various voltages, frequencies, and flow rates. A CCD camera with 5× objective was used during experiments to capture and record videos and images of the microfluidic channel. An AC signal with 20 Vpp (peak-to-peak) and 1 MHz, where yeast cells experience pDEP, was applied as the initial input signal. 

### 2.6. Finite Element Simulation

**Figure 6:**
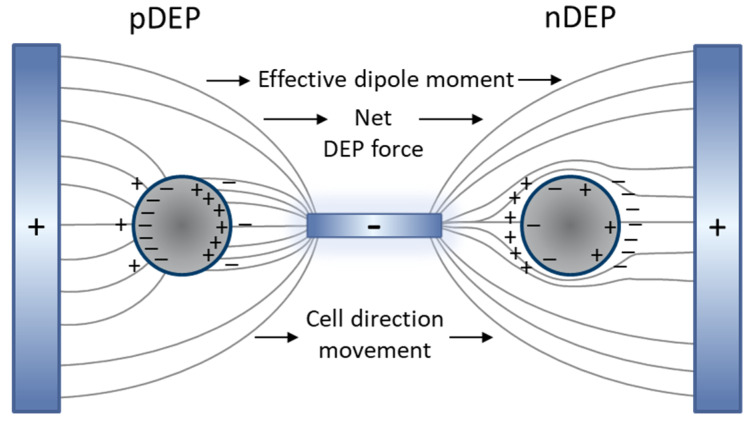
The induced dipole of the cell and medium with the presence of the non-uniform electric field and generation of pDEP and nDEP.

When an external electric field is imposed on the fluidic medium and suspending cells, medium and cells are being polarized. As shown in Figure 6, a net DEP force is induced in the direction of the high electric field intensity as a result of the non-uniform electric field distribution. 

Since this force is unique for every biological or nonbiological particle and exploits the differences in their dielectric properties, it can be used for characterization and manipulation of the cells in a fluidic medium. The time-dependent DEP on a cell in an inhomogeneous and time-varying electric field is proportional to the volume of the cell, as shown in the following equation [50]:

(1) $$F_{DEP}\left(t\right)=2\pi \varepsilon_{m}r^{3}\;Re\left[f_{CM}\right]\nabla E_{rms}^{2}$$

where $\varepsilon_{m}$, is the fluidic medium permittivity, *r* is particle radius, $E_{rms}^{\;}$ is the root-mean-square of the electric field strength and $Re\left[f_{CM}\right]$ is the real part of the Clausius-Mossotti (CM) factor as defined in the equation below [50]:

(2) $$f_{CM}=\frac{\varepsilon_{\;}{}_{c}^{*}-\varepsilon_{\;}{}_{m}^{*}\;}{\varepsilon_{\;}{}_{c}^{*}+2\varepsilon_{\;}{}_{m}^{*}}$$

(3) $$\varepsilon_{\;}{}_{\;}^{*}=\varepsilon -i\frac{\sigma }{\omega }$$

where $\varepsilon_{\;}{}_{c}^{*}$ and $\varepsilon_{\;}{}_{m}^{*}$ are the complex permittivity of the cell and the suspending medium, respectively. Complex permittivity is a function of conductivity (σ) and angular frequency of the applied electric field (ω). The $f_{CM}$ of biological cells, such as yeast cells in this study, can be evaluated by modeling concentric layers with different dielectric properties [50]. Based on Equations (2) and (3), $f_{CM}$ is a frequency dependent parameter and a function of relative magnitude of the cell with respect to its medium [50]. When the cell is more polarizable than the medium ($Re\left[f_{CM}\right]>0$), positive DEP (pDEP) moves the cells towards the maximum electric field intensity locations. When the cells are less polarizable than the medium ($Re\left[f_{CM}\right]<0$), they experience negative DEP (nDEP) which pushes them towards the zones of minimum electric field intensity. 

**Figure 7:**
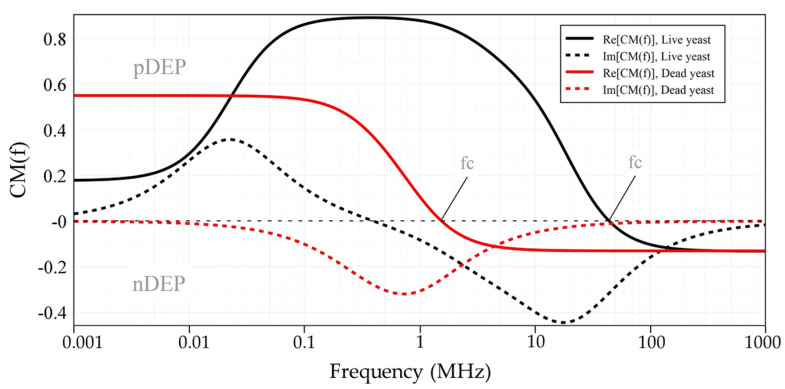
The Clausius-Mossotti factor of live and dead yeast cells suspended in DI-water as a function of frequency, using a two-shell model with the yeast parameters listed in Table 2.

The $f_{CM}$, as a function of the electric field frequency, for both live and dead yeast cells suspended in DI-water was numerically calculated using MATLAB and myDEP software [51], based on the two-shell model [50], where cells are assumed to possess two concentric layers of various electric and dielectric properties, as shown in Figure 7. Table 2 represents the dielectric values of yeast cells [50], and DI-water used for simulations. For live cells, the real part of the CM factor is bounded between 0.9 and ~−0.2. For dead cells this value is bounded between ~0.6 and ~−0.2. Variation of the applied signal frequency to the electrodes gives rise to DEP force in two opposite directions, which results in pDEP and nDEP. For the frequency ranges below crossover frequency (fc), $Re\left[f_{CM}\right]\;$is positive, while for higher frequencies $Re\left[f_{CM}\right]\;$is negative. The transition from the pDEP (top half) to nDEP (bottom half) which occurs at around 45 MHz and 1.45 MHz for live and dead yeast, respectively, is called crossover frequency (fc). This is a specific frequency at which the intrinsic properties of cells can be defined. 

**Figure 8:**
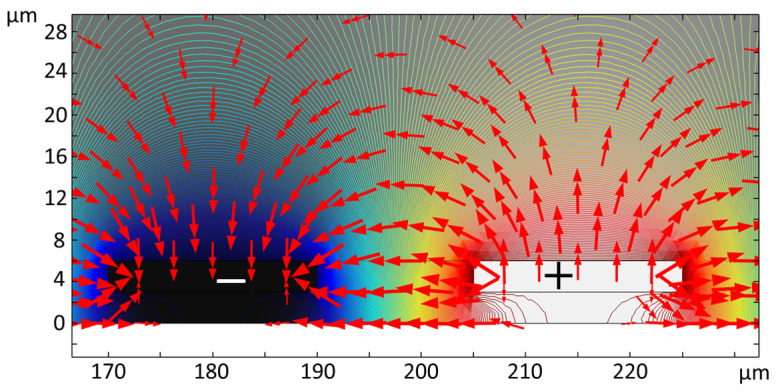
Numerical simulation results for the electric potential applied (line contour) to the IDEs and the electric field distribution (red arrows).

Several IDE structures were modeled in COMSOL 5.5 using a 2D model [47]. Using this model, the trajectory of the live and dead yeast cells through the microfluidic channel was simulated and the capabilities of different IDEs for cell immobilization were evaluated. Predictions from the developed simulations were compared with the experimental results. Figure 8 shows the electric potential contours (lines) imposed on the IDEs and the electric field distribution (arrows) over the electrodes in the channel. The electric field is intensified between IDE fingers and maximized at the rectangular corners of the electrodes [47]. This results in the non-uniform distribution of the electric field. The magnitude of the electric field over the IDEs decays with the distance over the IDEs towards the top of the microfluidic channel. 

## 3. Results

### 3.1. Dielectrophoretic Immobilization of Living Yeast Cells

**Figure 9:**
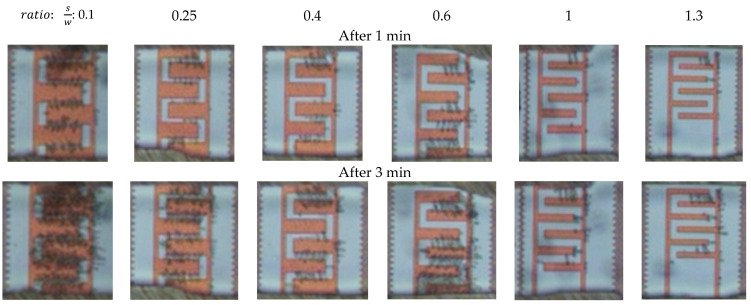

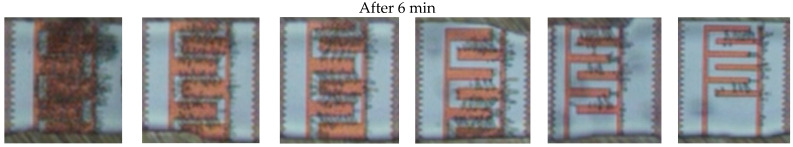
Micrographs of the immobilized yeast cells as a function of IDEs geometry (spacing to width ratios) at three time intervals of 1 min, 3 min, and 6 min. Immobilization conditions: 20 Vpp, 1 MHz, 1 µm·s^−1^ flow rate.

**Figure 10:**
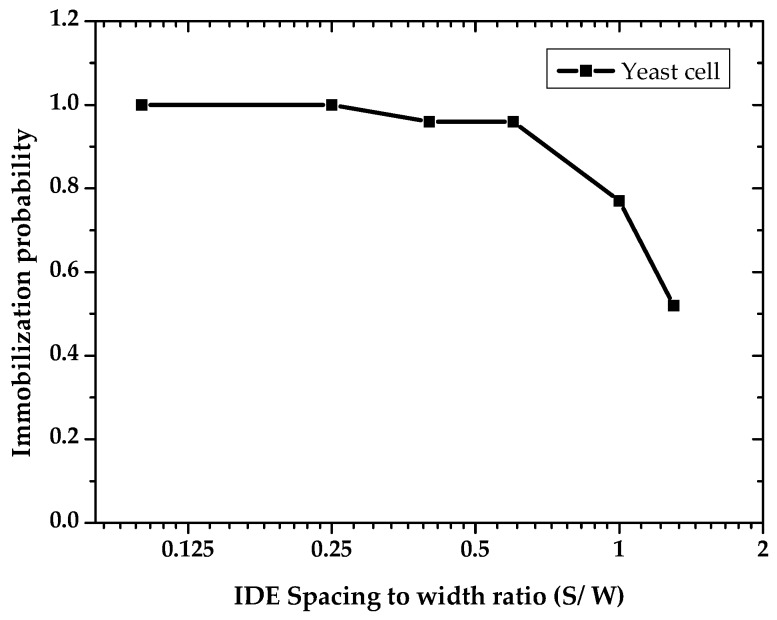
Calculated impact of IDE’s spacing to width ratio on the immobilization of yeast cells. Finite element modeling (FEM) for the dielectrophoretic immobilization of yeast cells was performed at 20 Vpp and 1 MHz.

After applying 20 Vpp at 1 MHz, obvious cell immobilization was observed. Cell entrapment started a few seconds after cell suspension had reached the IDEs. Trapping started at the electrode edges, where the electric field gradient intensity was increased. Figure 9 demonstrates the cell trapping performance of different IDE geometries at three time intervals. Cell polarization effect in a non-uniform electric field led to dipole-dipole interaction and forming of pearl chains of cells [21]. As shown in Figure 9, the number of immobilized yeast cells is strongly dependent on the geometrical ratio of the IDEs. The number of immobilized yeast cells is reduced with increasing IDEs geometrical ratio. Cell entrapment reaches its highest efficiency by using IDEs with the largest finger width (45 µm) and smallest gap spacing (5 µm) between adjacent fingers. Furthermore, immobilization of cells using IDEs with higher geometrical ratios is challenging. This is due to the fact that the entrapped cells desorb from the IDEs with greater S/W ratios throughout the immobilization process. In addition, it is observed that increasing an electrode width, with a constant spacing size, expand the number of entrapped cells. These optical observations are in line with the simulation results presented in Figure 10. Finite element modeling (FEM) simulations support the impact of geometrical parameters on the DEP effect. The probability of immobilizing cells increases with reduced geometrical (spacing to width) ratios of the IDEs. Figure 10 illustrates the impact of various geometry ratios on the immobilization probability (IP) of yeast cells. The immobilization probability is defined by the number of trapped cells to the total number of cells suspended in the fluidic medium.

**Figure 11:**
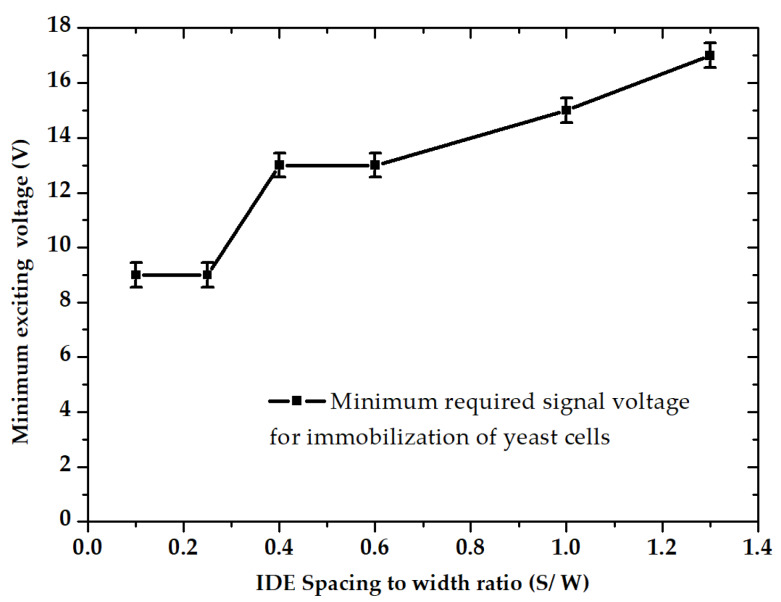
Minimum DEP driving voltage for the immobilization of yeast cells as function of spacing to width ratio, according to experimental data.

Increasing the IDEs ratio reversely impacts the IP. Furthermore, it can be seen that by keeping the spacing constant at 20 µm, there has been a steady decline in IP with decreasing width (at ratios of 0.6, 1, and 1.3). Therefore, it can be concluded that the DEP efficiency is highly influenced by IDE’s dimensional ratio (S/W). As illustrated in Figure 11, experimental results indicate that smaller IDE ratios reduce the required peak voltages for DEP immobilization.

For the largest IDE dimensional ratio, the required voltage values were roughly twice as much as the required value for the smallest IDE ratio. In addition, cell trapping was increased drastically with an increasing voltage trend. By increasing the gradient of the electric potential, the DEP force was raised and thus more cells were attracted to the electrodes. 

**Figure 12:**
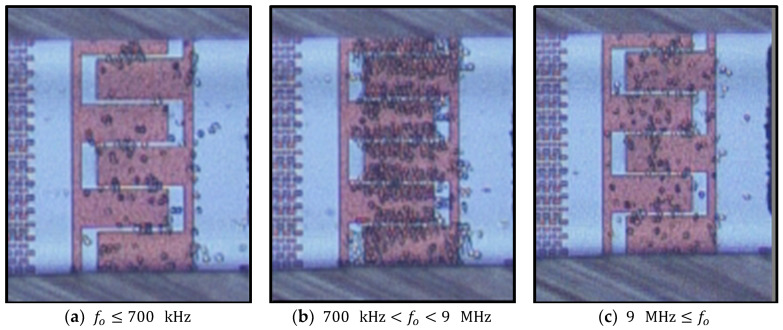
Frequency dependency of cell immobilization at 20 Vpp and a flow rate of 1 µm s^−1^. (**a**) High desorption rate of immobilized yeast cells; (**b**) Very high cell immobilization rate and very low desorption rate of immobilized cells; (**c**) High desorption rate of immobilized cells.

According to the experimental results, live cells can be trapped in a frequency range between 700 kHz and 9 MHz. Using frequencies above 10 MHz and below 300 kHz cells experience a repulsive force, which results in significant desorption of the immobilized cells from the electrodes. Figure 12 shows the frequency dependency of cell entrapment at 20 Vpp. In the frequency range between 900 kHz and 6 MHz, desorption rates are very low. Desorption rates increase drastically at lower frequencies ($f_{o}\leq 300\;\mathrm{kHz}$) due to weak pDEP and at higher frequencies ($10\;\mathrm{MHz}\leq f_{o}$) due to nDEP.

**Figure 13:**
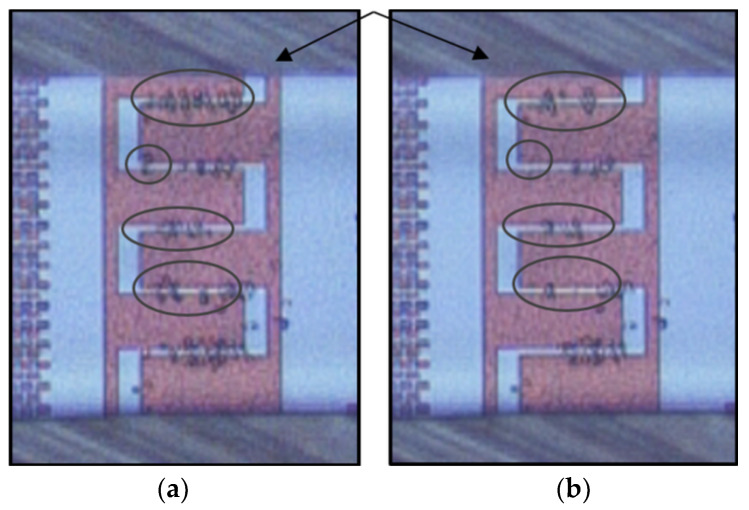
An example of weak immobilization at 8 MHz ($9\;V_{pp}$, and 1 μm·s^−1^): (**a**) Immobilization of cells after 2 min, and (**b**) 3 min, partial desorption of trapped cells from the IDEs.

At frequencies between 700 kHz and 900 kHz and between 7 MHz and 9 MHz, the immobilization stability of cells mainly dropped, and with the passage of the time immobilized cells gradually tend to desorb from the electrodes. Figure 13 illustrates the weak immobilization as a result of imposing an AC voltage of 9 Vpp at 8 MHz. 

The experimental results are in agreement with the simulation (see Figure 7), the yeast cells are forced by pDEP at frequencies lower than 10 MHz. However, fc was found to be 10 MHz, whereas the simulated prediction of fc is about 45 MHz. The large difference between the simulated and the experimentally evaluated values of fc could be caused by the simplicity of the used model in terms of cell wall, membrane, cytoplasm, and nucleus size.

### 3.2. Dielectrophoretic Separation of Live and Dead Yeast Cells

In order to differentiate between live and dead yeast cells, the impact of the AC frequency on the trajectory of live and dead cells was investigated experimentally and simulated using COMSOL. On the basis of the simulation shown in Figure 7, the DEP response of live and dead cells is significantly different at high frequencies [16]. Yeast cells subject to some modifications when they expose to heat shock. Such modifications could include a reduction in size and alterations to the dielectric properties of the cell. Due to the heat shock, intercellular water is reduced, and yeast cell experience water stress. This results in the wrinkling of the cell membrane and reduction in the cell diameter, which is associated with the shrinkage of the entire yeast cell [52,53,54]. The fc of dead yeasts occurs at ~1.45 MHz because dead cells lose their viability due to an impaired membrane. Their cytoplasmic conductivity is decreased while their membrane conductivity is increased. An impaired membrane of a dead cell polarized differently when it is exposed to an electric field. Thus, due to these dielectric discrepancies, responses of live and dead cells to the fringing electric field are dissimilar [16,21,55]. Our experimental results demonstrate that dead cells experienced an attractive force between 40 kHz and 1.45 MHz and can be trapped at the IDEs between 60 kHz and 1.45 MHz. At lower frequencies (<40 kHz), no DEP response was observed for dead yeasts. 

**Figure 14:**
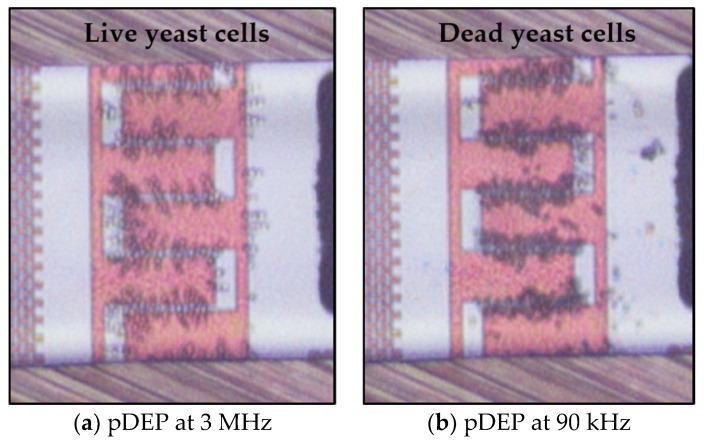
Separation of live and dead cells based on DEP characteristics. (**a**) Immobilize of live cells at the IDEs, while dead ones repel from the IDEs and leave the microfluidic channel at 20 Vpp, and (**b**) Immobilization of dead yeast cells at the IDEs).

Taking into account the distinct DEP responses of live and dead yeast cells at specific frequency ranges, preliminary demonstrations of the separation were performed. The concept was also simulated using COMSOL Multiphysics. Our simulation results were reasonably consistent with experimental results. Figure 14a shows a snapshot image for the separation of dead cells from live cells, where live cells immobilized at the IDEs at 3 MHz and 20 Vpp. In contrast, separation of live cells from dead ones, when dead cells immobilized at the electrodes at 90 kHz and 20 Vpp, is shown in Figure 14b. Therefore, separation of live and dead cells using the proposed method is achievable. Furthermore, the desorbed dead or live cells can be collected at the outlet of the microfluidic channel for further investigations and analyses.

**Figure 15:**
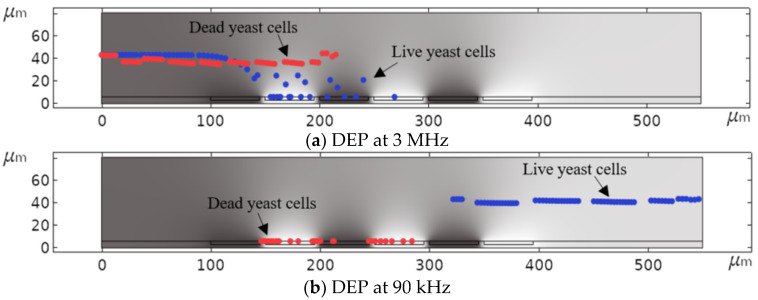
Simulated DEP separation of live and dead yeast cells: (**a**) Separation of dead cells from live cells, and (**b**) vice versa as a function of frequency.

Figure 15 demonstrates how DEP can be used diversely to isolate specific cells from a mixture of cells using the distinct DEP behavior of live and dead yeast cells. Keeping the signal frequency constant at 3 MHz, dead cells were separated from live cells, which were simultaneously immobilizing at the IDEs (Figure 15a). The opposite situation happens when a signal frequency in the range of$\;70\;\mathrm{kHz}\;\leq \;f_{o}\leq 100\;\mathrm{kHz}$ is applied to the system (Figure 15b). 

## 4. Conclusions

A silicon-based CMOS integrated microfluidic device for immobilization of live and dead yeast cells via DEP was investigated. The device has been used to differentiate between live and dead yeast cells based on the selective DEP forces, pDEP and nDEP. IDEs with various geometrical parameters were studied. The effect of DEP force on the trajectory of yeast cells as functions of voltage, frequency, flow-rate, and IDE geometry was studied experimentally. Besides, finite element modeling was used to predict the trajectories of the cells. Experimental and simulation results demonstrate that based on the specific properties of cells, the microfluidic device can be used to immobilize and separate specific cells by varying the AC frequency. It was found that the experimental results are in agreement with the simulation. 

## Figures and Tables

**Table 1:** Parametrical geometries of the IDEs, chosen based on the results reported in [49].

IDE Structure	IDE 1	IDE 2	IDE 3	IDE 4	IDE 5	IDE 6
S/W ratio	0.1	0.25	0.4	0.6	1	1.3
Spacing between finger (S)	5 (µm)	10 (µm)	15 (µm)	20 (µm)	20 (µm)	20 (µm)
IDE finger width (W)	45 (µm)	40 (µm)	35 (µm)	30 (µm)	20 (µm)	15 (µm)

**Table 2:** Yeast cell [50] and DI-water dielectric properties.

MUT ^1^	Permittivity			Conductivity (S/m)		
Di-water	78			1 × 10^−3^		
**Yeast**	**cp ^2^**	**cm ^3^**	**cw ^4^**	**cp ^2^**	**cm ^3^**	**cw ^4^**
Live yeast cell	50	6	60	0.2	2.5 × 10^−7^	1.4 × 10^−2^
Dead yeast cell	50	6	60	7 × 10^−3^	1.6 × 10^−3^	1.5 × 10^−3^

^1^ Material under test. ^2^ Cytoplasm. ^3^ Cell membrane. ^4^ Cell wall.
